# Multiscale Kinetic Monte Carlo Simulation of Self-Organized Growth of GaN/AlN Quantum Dots

**DOI:** 10.3390/nano12173052

**Published:** 2022-09-02

**Authors:** Jorge A. Budagosky, Alberto García-Cristóbal

**Affiliations:** 1 Nanotechnology on Surfaces and Plasma Group, Materials Science Institute of Seville (CSIC-US), C/ Américo Vespucio 49, 41092 Seville, Spain; 2 Instituto de Ciencia de los Materiales (ICMUV), Universidad de Valencia, C/ Catedràtic José Beltrán 2, 46980 Paterna, Spain

**Keywords:** kinetic Monte Carlo, heteroepitaxy, Stranski-Krastanov growth mode, strain relaxation, III-N semiconductors, gallium nitride, nucleation, self-organized quantum dots, 64.75 Yz, 68.55 A-, 77.80 bn, 77.84 Bw, 81.16 Rf

## Abstract

A three-dimensional kinetic Monte Carlo methodology is developed to study the strained epitaxial growth of wurtzite GaN/AlN quantum dots. It describes the kinetics of effective GaN adatoms on an hexagonal lattice. The elastic strain energy is evaluated by a purposely devised procedure: first, we take advantage of the fact that the deformation in a lattice-mismatched heterostructure is equivalent to that obtained by assuming that one of the regions of the system is subjected to a properly chosen uniform stress (Eshelby inclusion concept), and then the strain is obtained by applying the Green’s function method. The standard Monte Carlo method has been modified to implement a multiscale algorithm that allows the isolated adatoms to perform long diffusion jumps. With these state-of-the art modifications, it is possible to perform efficiently simulations over large areas and long elapsed times. We have taylored the model to the conditions of molecular beam epitaxy under N-rich conditions. The corresponding simulations reproduce the different stages of the Stranski–Krastanov transition, showing quantitative agreement with the experimental findings concerning the critical deposition, and island size and density. The influence of growth parameters, such as the relative fluxes of Ga and N and the substrate temperature, is also studied and found to be consistent with the experimental observations. In addition, the growth of stacked layers of quantum dots is also simulated and the conditions for their vertical alignment and homogenization are illustrated. In summary, the developed methodology allows one to reproduce the main features of the self-organized quantum dot growth and to understand the microscopic mechanisms at play.

## 1. Introduction

Semiconductor heteroepitaxy involves, in most cases, a lattice mismatch between the epitaxial layer and the substrate that results in the appearance of strain in the system. Such strained heteroepitaxy is an area of materials research of enormous interest because of both its exploitation in a variety of electronic and optoelectronic devices and its academic value as a playground for the study of the interplay of strain-induced thermodynamical and kinetic effects [1]. One key issue with such heteroepitaxy is that the strain introduced must always find a way to be relieved as the thickness of the epitaxial film increases. In the so-called Stranski–Krastanov (SK) growth mode, this strain relief occurs, after a critical thickness is reached, by elastic relaxation of the stressed film via the formation of coherent three-dimensional (3D) nanometric islands [1]. It was recognized very early that, if the materials are appropriately selected, the overgrown structures can accommodate a few confined electronic states, and therefore can be used as quantum dots (QDs) [2]. Because of its origin, they are often referred to as self-organized or self-assembled quantum dots. One advantage of this approach is the ease of the fabrication of high density ensembles of QDs, a necessary condition for their use in optoelectronic devices [2]. On the other hand, due to the stochastic nature of the growth process, the resulting islands present a certain size and position dispersion. This inhomogeneity makes difficult to control the desired properties necessary for device implementation. The most studied examples of strained heteroepitaxy are the InAs/GaAs and Ge/Si systems (with lattice mismatch of 6% and 4%, respectively), but there are many more combinations of potential technological interest, such as InAs/InP, CdSe/ZnSe, PbSe/PbTe, and GaN/AlN, to name a few.

The parameters and mechanisms that underlie the self-assembly of 3D islands have long been the subject of great interest. From the experimental point of view, the goal has been to master the specific conditions that allow to select the growth mode, and provide control over the size and density of the islands. In this respect, experimental data indicate that the growth of self-organized QDs is sensitive to parameters such as substrate temperature, fluxes of the constituent materials, growth interruption time, etc. Although these studies provide a valuable empirical knowledge, the microscopic origin of the established general trends is not fully understood, owing to the complexity of the physics of strained layer growth. A complementary approach is the development of numerical simulations. At present, the most common and versatile growth modeling strategy is based on the kinetic Monte Carlo (KMC) method. Its advantage lies in that, due to its stochastic nature, it is able to cover the experimentally relevant growth times and system sizes, while having access in principle to the full atomistic structure. Since the growth of self-assembled QDs in the SK mode is driven by the elastic relaxation, the incorporation of the strain in the KMC methodology is mandatory. It has to be noted though that the computational cost in simulating epitaxial systems with strain is more expensive than for systems without strain because the elastic displacement field has to be dynamically updated following the changes in the growing film. The KMC method has been implemented with a varying degree of sophistication for the simulation of self-assembled epitaxial growth [3,4,5,6,7,8,9,10].

Most of the existing work on the growth of self-assembled QDs, both experimental and simulation, is devoted to the study of InAs/GaAs and Si/Ge systems with cubic crystalline structures. There has followed, however, a remarkable progress with the strained heteroepitaxial growth of group-III nitrides with wurtzite structure, which have a wide range of applications in the field of optoelectronics and high power electronics [11]. The GaN/AlN heterostructure, normally grown along the polar axis of the crystal structure, is a typical example; it is characterized by a lattice mismatch of the order of 2.4% and has been demonstrated to present a SK growth transition leading to wurtzite GaN QDs. In the literature, GaN/AlN quantum dots have been obtained by three major epitaxial growth methods: plasma-assisted molecular beam epitaxy [12], ammonia-assisted molecular beam epitaxy [13], and metalorganic vapor-phase epitaxy [14,15]. In this paper, we focus on the GaN/AlN QDs obtained by plasma-assisted molecular beam epitaxy (PA-MBE) at CEA Grenoble, since their growth has been thoroughly analyzed by a range of experimental techniques [12,16,17,18,19,20,21,22,23,24,25]. Concerning the KMC simulations, there exist just a few studies devoted to the growth of III-N wurtzite-type materials. They are, however, mainly focused on GaN homoepitaxy [26,27,28,29,30,31]. To the best of our knowledge, nobody has reported KMC simulations of self-organized growth of III-N QDs so far.

In this paper, we propose a 3D lattice kinetic Monte Carlo model to study the growth of self-organized GaN/AlN QDs. Our aim is to perform simulations on length scales close to one hundred nanometers and time scales of tens of seconds, by including the evolving strain distribution in the system. This goal poses a strong computational challenge that we have tackled by: (i) simulating effective GaN adatoms, instead of following in detail the separate behavior of real Ga and N atoms, (ii) implementing a fast multiscale algorithm that allows the adatoms to perform long diffusion jumps across multiple sites [32], and (iii) developing an efficient procedure to obtain the elastic strain energy distribution based on continuum elasticity theory. In order to validate the proposed model, we have tailored it for its comparison against the experimental resuls on GaN/AlN QDs grown by PA-MBE under N-rich conditions. The corresponding simulations of the evolution of the surface morphology reproduce the different stages of the SK transition from an initial two-dimensional (2D) layer to the full development of 3D QDs, and demonstrate quantitative agreement with the experimental findings concerning the critical deposition, and island size and density. The influence of relevant growth parameters, such as growth temperature and the relative fluxes of Ga and N, is also studied and found to be consistent with the experimental observations. Additionally, we have briefly simulated the growth of multiple stacked QD layers, and demonstrated how the strain field can lead to both vertical alignment and increase the uniformity of the QD ensembles of the upper layers. These findings are in qualitative agreement with the reported experimental results [22].

The paper is organized as follows. In Section 2, we describe the theoretical background and the procedure used in our simulations. In Section 3, we discuss the results obtained, including a comparison with available experiments. Finally, Section 4 contains the overall discussion and the conclusions extracted from the work.

## 2. Description of the Modeling Strategy

This section describes the specifics of the lattice kinetic Monte Carlo (KMC) method that we have implemented for the simulation of epitaxial growth of wurtzite GaN self-assembled quantum dot layers on (0001)AlN surfaces. First, we set up the model geometry and comment on the selected events and relevant parameters, as well as the technical solutions we have adopted to accelerate the computations. Afterwards, we present the procedure followed to calculate and incorporate the strain energy of the lattice-mismatched system into the heteroepitaxial growth simulations.

### 2.1. KMC Model and Implementation

**Figure 1:**
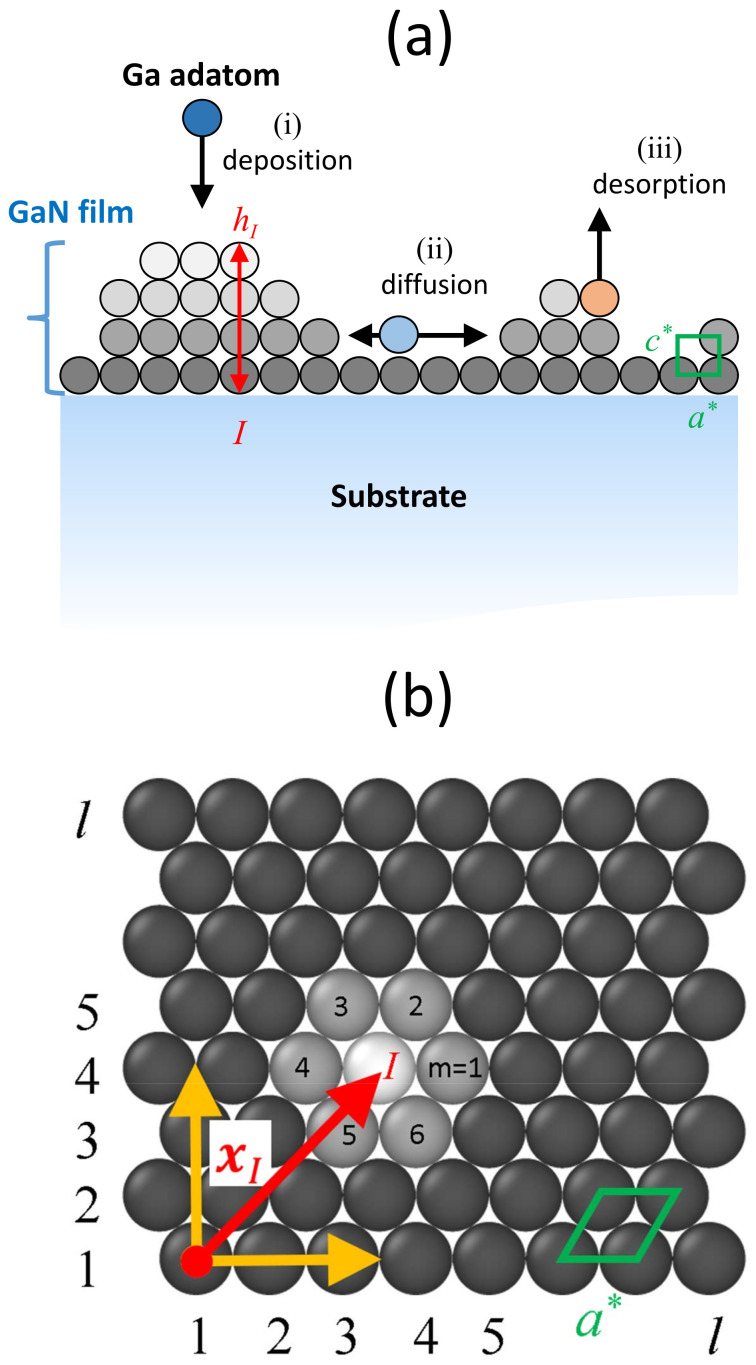
(**a**) Cross section of the system under study. The actual domain of simulation is the indicated GaN film, modeled as a set of adatoms located at predefined lattice sites, and represented here as spheres in hexagonal unit cells of parameters $a^{*}$ and $c^{*}$. The in-plane sites are labeled by integers *I*, and the corresponding local heights are denoted by $h_{I}$ (see text). Also sketched are the possible processes that a given adatom can experience: (i) deposition, (ii) diffusion, and (iii) desorption. (**b**) Top view of the GaN film, with indication of the 2D hexagonal lattice, and illustration of the possible diffusion jumps that an adatom at $x_{I}$ can make.

The system under study consists of a GaN thin film growing on a substrate. The substrate plays a passive role here, not being affected by the phenomena taking place at the surface. Therefore, the simulation domain reduces in practice to the deposited GaN film. In principle, two separate atomic species, Ga and N, should be simulated explicitly within the KMC model. Such a detailed description of all possible processes would be challenging, but one aspect of GaN growth provides a simplification. Under sufficiently N-rich conditions to insure microscopic stoichiometry, the Ga adatom kinetics, rather than the Ga-N reaction kinetics, is the rate-limiting step. The GaN growth can thus be effectively regarded as a single species process involving the attachment and migration of Ga adatoms. Although the N kinetics is not considered explicitly in this approach, it will be taken care of in an averaged way by using effective parameters for the Ga adatom diffusion rate that depend on the Ga/N flux ratio. For this reason, we use the term “effective GaN adatoms” to refer to the simulated species. The GaN film is thus modeled as a set of effective GaN adatoms located at the sites of a hexagonal lattice of parameters $a^{*}$ and $c^{*}$; see Figure 1. The in-plane projection of the domain, shown in Figure 1b, has *l* sites on each side, so that the total number of two-dimensional (2D) lattice sites is $N_{l}=l\times l$, and the simulated surface is $S=N_{l}\Delta x_{1}\Delta x_{2}$, where $\Delta x_{1}=a^{*}$ and $\Delta x_{2}=a^{*}\cos\left(\pi /6\right)=\sqrt{3}a^{*}/2$. Solid-on-solid restrictions are imposed (i.e., lateral overhangs and vacancies are not allowed) and periodic boundary conditions are applied in both lateral directions. The GaN adatoms of our simulation distribute dynamically over this lattice as explained below. It should be noted though that our model is not strictly atomistic. The consideration of effective GaN adatoms hopping on a lattice and the use of the effective parameters introduced below have to be understood as a mean-field approach [33,34]. In all simulations we used $c^{*}=c_{\mathrm{AlN}}/2$, where $c_{\mathrm{AlN}}=0.4982$ nm is the axial lattice parameter of the substrate wurtzite structure ($c^{*}$ being therefore the thickness of one monolayer, ML). The effective in-plane parameter is taken to be $a^{*}=0.502$ nm. Our model does not contemplate the occurrence of substrate surface reconstruction, vacancies or intermixing, which nevertheless are thought to play a minor role in the growth of GaN self-assembled quantum dots.

The instantaneous configuration of the film can be specified by an occupation array ${\left\{\sigma_{I}\right\}}_{I=1}^{N_{l}}$, where *I* is a suitably defined integer index running over all 2D lattice sites, and $\sigma_{I}$ represents the number of adatoms piled up at site *I*. The local thickness of the GaN layer at each site *I* is then $h_{I}=\sigma_{I}c^{*}$, see Figure 1a, and is used below to represent the surface morphology. For later reference, we denote by $x_{I}$ the in-plane position vector of 2D lattice site *I*. Within the KMC method, the configuration of the GaN film evolves in time by stochastic changes of the occupation of the discrete lattice sites: In its standard version, at each time step, upon the random selection of a site, a properly selected process (among a catalog of possible events, see below) is performed there, and the configuration array $\left\{\sigma_{I}\right\}$ is updated accordingly. The corresponding time interval is calculated and added to the elapsed simulation time [35,36].

For the simulation of the epitaxial growth we have included three types of processes: deposition (adsorption), surface diffusion, and evaporation (desorption), which are schematically indicated in Figure 1a. Each elementary event is associated with a rate, as follows:

(i) During the growth simulation, the adatoms are randomly deposited onto the surface, with a deposition rate $r^{\mathrm{dep}}=FN_{l}$, where *F* represent the deposition flux (in ML/s).
(ii) The adatoms sitting on the surface are allowed to randomly hop to one of its six nearest neighbor sites, see Figure 1b, with a jump rate defined by an Arrhenius-type expression:
  (1) $$r_{\mathrm{dif}}=\nu_{0}\;\exp\left(-\frac{E_{\mathrm{dif}}}{k_{B}T}\right)\;,$$
  where $E_{\mathrm{dif}}$ is the diffusion energy barrier, $k_{B}$ the Boltzmann constant, and *T* the substrate temperature. The factor $\nu_{0}$, called attempt frequency, defines the time scale of the surface processes, and is typically related with the vibrational frequency of a surface adatom. Here, we take $\nu_{0}=10^{13}$ s${}^{-1}$.
(iii) The adatom desorption process is analogously described by the rate:
  (2) $$r_{\mathrm{des}}=\nu_{0}\;\exp\left(-\frac{E_{\mathrm{des}}}{k_{B}T}\right)\;,$$
  where $E_{\mathrm{des}}$ is the corresponding energy barrier.

The energy barriers of both diffusion and desorption processes are actually composed of various contributions:

(3a) $$\begin{array}{cc} E_{\mathrm{dif}} & =E_{\mathrm{dif}}^{\left(0\right)}+nE_{b}+mE_{\mathrm{step}}-E_{\mathrm{str}}\;, \end{array}$$

(3b) $$\begin{array}{cc} E_{\mathrm{des}} & =E_{\mathrm{des}}^{\left(0\right)}+nE_{b}-E_{\mathrm{str}}\;. \end{array}$$

The terms $E_{\mathrm{dif}}^{\left(0\right)}$ ($E_{\mathrm{des}}^{\left(0\right)}$) are the contribution to the diffusion (desorption) barriers due to the binding to the underlying surface. For both diffusion and desorption barriers we assumed a simple bond-counting rule, where the energy $E_{b}$ is a measure of the binding energy between lateral neighbor adatoms. Accordingly, in case a given adatom is bonded to *n* neighbors, an increase $+nE_{b}$ of the diffusion and desorption barriers is introduced. In the case of the hexagonal lattice, *n* runs from 0 (isolated adatom) to 6 (completely surrounded adatom). In some instances, the selected diffusion jump would take place across a single step, i.e., between sites with a difference in height of $c^{*}$, as would occur at an island edge. Single jumps across multiple steps are explicitly forbidden in our model. In the single step jumps one has to take into account the (Ehrlich-)Schwöbel effect [30,37,38,39]. This is effectively accounted for here by adding a term $+mE_{\mathrm{step}}$, where $E_{\mathrm{step}}$ is the magnitude of the Schwöbel barrier that the adatom has to surmount to cross the step edge, and *m* is the number of in-plane jump directions in which there is a step. For simplicity, in our simulations the additional step barrier is only included for downward step jumps. In the growth of a material lattice-mismatched to the substrate (as in the case of GaN on AlN), the growing layer is (inhomogeneously) strained and there is a corresponding accumulation of elastic energy. Consequently, the surface adatoms will diffuse (desorb) more often from the strained sites than from the relaxed ones [40]. As time passes by, this will lead to a net flux of adatoms from the strained to the relaxed areas, which is at the origin of the self-assembly of islands in these heteroepitaxial systems. To incorporate this effect into our simulation procedure, we introduce a strain-induced barrier term $-E_{\mathrm{str}}$, reflecting the increased likelihood of diffusion and evaporation events at sites where $E_{\mathrm{str}}$ is larger. The calculation of $E_{\mathrm{str}}$ will be discussed in Section 2.2. We note that both $E_{\mathrm{dif}}$ and $E_{\mathrm{des}}$ actually depend on the local environment (through *n* and *m*) and absolute position (through space-dependent $E_{\mathrm{str}}$) of the surface adatom considered.

During the KMC simulations, the system just described is evolved in time by using an event-based Bortz–Kalos–Lebowitz (BKL) algorithm [32,41]. The corresponding time interval Δt is calculated and added to the elapsed simulation time. In this work, compelled by the general requirement to increase the efficiency of growth simulations in large surfaces and for sufficiently long times, and the specific need to include in a simple way the effect of strain on the surface kinetics, we have incorporated the following modifications over the standard BKL algorithm:

- At every time step, the systematic search across the entire surface for the sites attainable by the process selected by the BKL algorithm has a cost in CPU time scaling with the surface size. To avoid this problem, we have used an inverted-list algorithm [42]. Since the updating of the inverted lists is a local procedure, the adatom search process becomes now independent of the surface size. This approach is specially efficient in the case of having a not too extensive catalog of possible events. In our case, in the absence of Schwöbel and strain-induced barriers, the system would have a very simple rate structure: There are only seven transitions for the diffusion and desorption processes, corresponding to the possible bonds to nearest neighbors (n=0,…,6). To include the (Ehrlich-)Schwöbel effect (in cases where the jump occurs across a step) and the inhomogeneous strain-induced barrier $E_{\mathrm{str}}$, without having to modify the structure of inverted lists mentioned above, we use an acceptance-rejection algorithm [42].
- In growth regimes where surface diffusion plays a dominant role, most of the computation time is spent in calculating the adatom diffusion random walks. However, the associated jump rates for isolated and bonded adatoms can differ by orders of magnitude, due to the relative factor $\exp\left(-\frac{nE_{b}}{k_{B}T}\right)$. We use the multiscale kinetic Monte Carlo method proposed in Ref. [32] that takes advantage of this disparity in the adatom dynamics by allowing the isolated adatoms to make longer jumps than the bonded ones. This reduces drastically the number of Monte Carlo steps required to complete the simulation. For example, for a surface with $N_{l}=500\times 500$ lattice sites, a tenfold reduction in computation time can be easily achieved. The long jumps in the multiscale model are an effective way of simulating by a single event a chain of multiple short jumps. We mentioned earlier that the presence of a nonuniform surface elastic energy is expected to lead, for sufficiently long times, to a preferential diffusion towards more relaxed regions. Therefore, it is natural to introduce, for long jumps (with associated long elapsed times), a *bias* in the jump direction that gives preference to the arrival sites with the lowest values of $E_{\mathrm{str}}$. In this work, we have implemented that idea as follows: Once an isolated adatom (n=0) has been selected, we scan its neighborhod in search of obstacles (steps to upper terraces, other isolated adatoms or clusters). This search sets the number of sites *L* (>1) for the long jump, and the jump direction α (α=1,⋯,6) is chosen according to the following probability distribution:
  (4) $$p_{\alpha }=w_{\alpha }/\sum_{\beta =1}^{6}w_{\beta }\;,$$
  with
  (5) $$w_{\alpha }=\exp\left(-\frac{\eta_{\alpha }E_{\mathrm{step}}+k\left(L-1\right)\Delta E_{\mathrm{str}}^{\left(\alpha \right)}}{k_{B}T}\right)\;,$$
  where the term $\eta_{\alpha }E_{\mathrm{step}}$ accounts for the possible presence of Schwöbel barriers: $\eta_{\alpha }=1$ ($\eta_{\alpha }=0$) if there is (there is not) a step when making a jump of *L* sites along direction α. The strain-induced bias term $k\left(L-1\right)\Delta E_{\mathrm{str}}^{\left(\alpha \right)}$ is proportional to the elastic energy difference $\Delta E_{\mathrm{str}}^{\left(\alpha \right)}$ between the target site, located *L* sites away along direction α, and the initial site. The factor $\left(L-1\right)$ introduces an explicit dependence of the bias on the jump length *L*. The coefficient *k* is a free input parameter of the model. A similar parameter was used in Ref. [9] to simulate superlattices of PbSe/PbEuTe QDs. In our case, the optimum value k=12 was determined so that the simulations reproduce the general features of growth experiments in GaN/AlN systems.

In principle, one could try to determine the parameters of the KMC model on the basis of the energetics obtained from ab initio total energy calculations [29,43]. However, this is outside the scope of this work, and we will instead estimate the strain-independent model parameters ($E_{\mathrm{dif}}^{\left(0\right)},E_{\mathrm{des}}^{\left(0\right)},E_{b},E_{\mathrm{step}}$) by trying to reproduce various well established features of the homoepitaxial growth of GaN (see Section 3.1).

Since this work is ultimately interested in the growth of QDs in the Stranski–Krastanov mode, all the simulations begin with a first monolayer of GaN already formed. The output of the simulation at later times is a certain array $\left\{h_{I}\right\}$, which defines the inhomogeneous thickness of the GaN film. The simulations can be analyzed qualitatively by direct observation of the resulting surface morphology, and quantitatively by computing, at selected stages of the simulation, quantities such as the dot density or the dot height and diameter distribution. A quantity of special interest that we extract from our simulations is the surface step density $S_{d}$ (associated to the degree of surface roughness), which has been demonstrated to provide a direct representation of the specular electron diffraction (RHEED) intensities recorded in a variety of growth experiments [33]. In the analysis of RHEED experiments that we present below, we have represented the quantity $1-S_{d}$ instead of $S_{d}$, since the maxima (minima) in the RHEED traces occur at situations when the density of steps at the surface of the GaN layer is minimal (maximal).

### 2.2. Calculation of the Strain-Induced Energy Barrier $E_{str}$

As advanced above, to simulate the growth process in GaN/AlN systems, due to the mismatch between the lattice parameters of GaN and AlN, it is necessary to take into account the strain field accumulated in the GaN epitaxial layer. Since morphological changes are restricted to the surface of the material, we focus here on the calculation of the elastic strain energy density at the free surface of GaN, which will enter into the KMC calculations through the strain-induced barrier $E_{\mathrm{str}}$.

**Figure 2:**
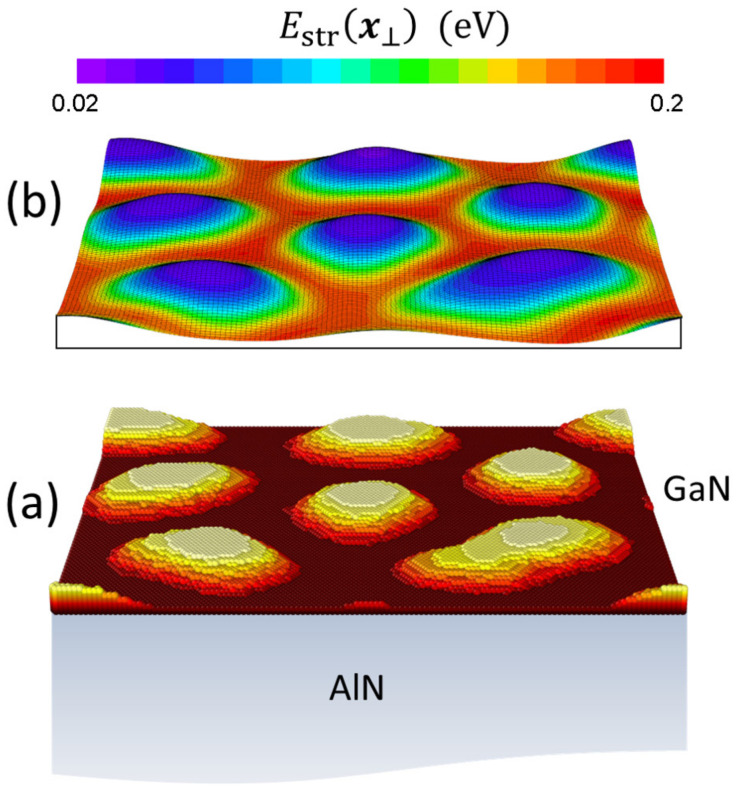
Schematic illustration of an epitaxial GaN layer on a semi-infinite AlN substrate. Both the original discrete description $\left\{h_{I}\right\}$ (**a**) and the smoothed version $h(x_{⊥})$ (**b**) of the surface morphology are shown. The topography in part (**b**) is overlaid by a color map representing the strain energy $E_{\mathrm{str}}\left(x_{⊥}\right)$, as calculated within continuum elasticity theory (see text).

At this point, there are alternative possibilities to calculate $E_{\mathrm{str}}$. One approach is based on the so-called ball-and-spring models, where an explicit physical displacement of the adatoms from their initial lattice site is allowed, and a system of restoring forces is implemented (off-lattice models) [10,44,45]. However, given the effective character of the adatoms in our model, there is not much sense in pursuing an atomistic description of the elastic energy. We have opted instead for another approach, in which the strain field in the growing layer+substrate system is treated in the framework of the continuum theory of elastic media, and afterwards used to determine $E_{\mathrm{str}}$. To couple the discrete nature of our original model to a continuous description of the system, it is necessary to define first a smoothed version of the surface that can be used in the calculation of the strain energy. To that end, we have used a coarse-graining strategy that uses an appropriate filter to obtain a function $h(x_{⊥})$ describing the continuous surface morphology from the values $\left\{h_{I}\right\}$ defining the GaN layer thickness in the original discrete description (see Figure 2a) [46].

Once $h(x_{⊥})$ is determined, the strain field at the surface and the corresponding strain energy density $U\left(x_{⊥}\right)$ are calculated in this paper as follows. The mismatched GaN layer (together with the buried QD layers, if present) is considered as an inclusion in the matrix given by the AlN substrate (see Figure 2b). According to Eshelby’s theory, the inclusion regions act as the source of the strain, which extends throughout the system [47]. For inclusions buried under a flat surface, the exact strain and stress distribution can in principle be obtained by using the Green’s function method [48]. However, in our case we face the need of implementing the stress-free boundary condition on the non-flat surface given by $h(x_{⊥})$. We have circumvented this problem by using an small-slope approximation of the GaN surface topography, in which it is exploited that $\left|\nabla_{⊥}h\left(x_{⊥}\right)\right|\ll 1$ to apply a perturbation procedure and simplify the problem [49]. Experimental observations generally show that the diameter of the GaN/AlN QDs exceeds by one order of magnitude their height, which justifies the use of this approach. The simplification conveyed by the perturbative treatment allows one to approximate the system with inhomogeneous thickness and stress-free boundary conditions by a system with a flat surface and a prescribed distribution of surface tractions, determined by $h(x_{⊥})$ and the lattice mismatch [49]. It is then possible to use the elastic Green’s function for a transversely isotropic half-space [50]. By following the sketched procedure, we can obtain the strain, stress, and strain energy density $U\left(x_{⊥}\right)$ at the surface of an arbitrary system of small-slope (GaN) islands on a (0001)-oriented semi-infinite (AlN) matrix. The details of our strain calculations will be discussed more extensively in a forthcoming publication [51]. The required parameters are the lattice mismatch of the GaN/AlN system, $\left(a_{\mathrm{AlN}}-a_{\mathrm{GaN}}\right)/a_{\mathrm{GaN}}=-0.024$ and $\left(c_{\mathrm{AlN}}-c_{\mathrm{GaN}}\right)/c_{\mathrm{GaN}}=-0.039$, and the AlN elastic constants.

Finally, we define the energy $E_{\mathrm{str}}\left(x_{⊥}\right)$ by:

(6) $$E_{\mathrm{str}}\left(x_{⊥}\right)=\Omega^{*}U\left(x_{⊥}\right)f\left(x_{⊥}\right)\;,$$

where $\Omega^{*}=\sqrt{3}/2\;{a^{*}}^{2}c^{*}$ is the volume of the unit cell occupied by each GaN adatom, and $f\left(x_{⊥}\right)$ is a function that heuristically takes into account the formation of the GaN wetting layer [52]:

(7) $$f\left(x_{⊥}\right)=1-\frac{1}{1+\exp\left[4\left(\frac{h\left(x_{⊥}\right)}{c^{*}}-\gamma \right)\right]}\;.$$

The parameter γ=1.5 has been chosen in order to obtain a critical thickness of 2 ML for the SK transition, as occurs in the growth experiments that we compare below with our simulations. In Figure 2b, we illustrate the calculation of $E_{\mathrm{str}}\left(x_{⊥}\right)$ for a typical situation. The discrete values of the strain-induced barrier energy appearing in the KMC simulations are then given by $E_{\mathrm{str}}\left(I\right)=E_{\mathrm{str}}\left(x_{⊥}=x_{I}\right)$.

Despite the efficiency of the procedure devised, the cost in CPU time to update the surface strain energy density of the film at each time step would still be prohibitive. However, major changes in the surface morphology occur at a time scale much greater than that of the individual surface events. Therefore, in all the simulations we have updated the strain-induced energy only at every $n_{\mathrm{str}}=500\left(l/64\right)$ time steps. Tests using lower values of $n_{\mathrm{str}}$ show no qualitative changes.

## 3. Simulation Results

In this section, we present the results of our numerical simulations. We first present a strategy to calibrate some of the parameters of the model by a comparison of the simulations with GaN homoepitaxial growth experiments. We will afterwards investigate the effect of some relevant parameters (GaN coverage Θ, flux ratio $\phi_{\mathrm{Ga}}/\phi_{N}$ and substrate temperature *T*) on the morphology of a single GaN QD layer grown on AlN. Finally, we will study the growth of stacks of GaN/AlN QD layers. All our simulations have been performed on a lattice of $N_{l}=l\times l=256\times 256$ sites (corresponding to an area of S∼128.4 nm ×111.2 nm).

### 3.1. Calibration of the Activation Energies by Homoepitaxial Growth Simulations

It is well established experimentally that the relative amount of Ga and N on the surface significantly influences the kinetics of MBE growth [20,21]. Although the details behind that effect cannot be explicitly taken into account in our effective KMC model, we explore here the possibility of indirectly accounting for it by proposing a dependence of the activation energies on the ratio between the Ga and N fluxes. However, due to the evaporation processes and decomposition of GaN at the high substrate temperatures at which MBE growth takes place, the effective fluxes $\left(\phi_{\mathrm{Ga}}^{\mathrm{eff}},\phi_{N}^{\mathrm{eff}}\right)$ are not necessarily equal to the nominal fluxes $\left(\phi_{\mathrm{Ga}},\phi_{N}\right)$ (the output fluxes of the Knudsen cells of the MBE system). In the N-rich regime that we are interested in, we can consider the N desorption as negligible, so that $\phi_{N}^{\mathrm{eff}}=\phi_{N}$, and the growth of GaN is limited by the net flux of Ga [20,21,53]. In Appendix A we propose a simple model to obtain $\phi_{\mathrm{Ga}}^{\mathrm{eff}}$ in terms of $\left(\phi_{\mathrm{Ga}},\phi_{N}\right)$, as well as the temperature, which is valid only in the N-rich regime. In the simulations in this paper we assimilate the effective flux $\phi_{\mathrm{Ga}}^{\mathrm{eff}}$ to the deposition flux *F* introduced in Section 2.1.

**Figure 3:**
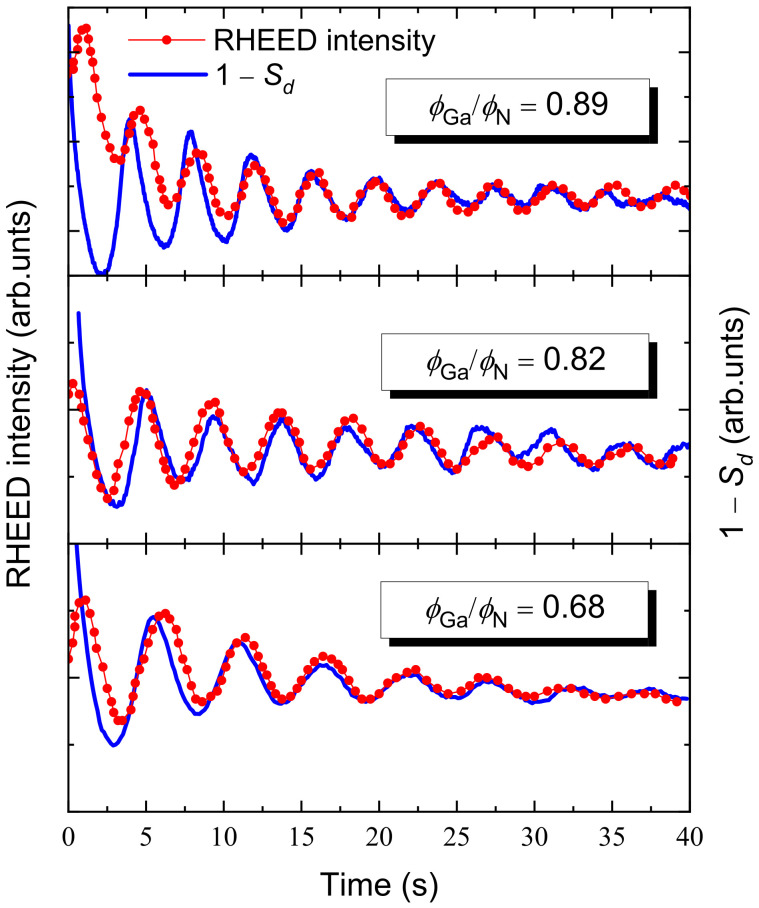
Comparison between the RHEED traces measured during GaN homoepitaxial growth at $T=730{\;}^{\circ }$C under different ratios $\phi_{\mathrm{Ga}}/\phi_{N}$ in Ref. [20] with the step density $1-S_{d}$ curves obtained from simulations with the fitted activation energies. In all cases, $\phi_{N}=0.28$ ML/s.

To obtain an estimate of the activation energies of our KMC model, we first assume them to be dependent on the effective flux ratio $\eta \equiv \phi_{\mathrm{Ga}}^{\mathrm{eff}}/\phi_{N}$ through appropiate model functions. The free parameters of these functions have been fixed by fitting the RHEED traces measured during GaN homoepitaxial growth [20] against the corresponding temporal evolution of $1-S_{d}$ calculated from our simulations. Figure 3 shows the RHEED traces for three nominal ratios $\phi_{\mathrm{Ga}}/\phi_{N}$ together with the time evolution of $1-S_{d}$ calculated with the fitted activation energies. Of course, in the simulations of homoepitaxial growth (in our case GaN on GaN), the term $-E_{str}$ is absent from the diffusion and desorption energy barrier expressions (Section 2.1). The approximate expressions for the activation energies that are obtained after this calibration procedure are:

(8) $$\begin{array}{ccc} E_{\mathrm{dif}}^{\left(0\right)} & ≃ & 1.78-4.87\times 10^{-3}\;e^{4\eta }\;\;\mathrm{eV}\;,\\ E_{b} & ≃ & 0.41-0.12\;\eta \;\;\mathrm{eV}\;,\\ E_{\mathrm{des}}^{\left(0\right)} & ≃ & 2.53-0.17\;\eta \;\;\mathrm{eV}\;,\\ E_{\mathrm{step}} & ≃ & 0.12-0.06\;\frac{\eta^{14.4}}{0.75^{14.4}+\eta^{14.4}}\;\;\mathrm{eV}\;. \end{array}$$

As we noted above, due to the simplicity of the growth model, the activation energies of the different processes should be regarded as effective parameters, i.e., parameters whose values have to be understood as averages of the microscopic processes that we are not considering explicitly.

### 3.2. Onset of the Stranski–Krastanov Growth Mode and Formation of Quantum Dots

In this section, we analyze the heteroepitaxial growth of a GaN film on an AlN substrate as a function of the coverage Θ (amount of deposited GaN). The substrate temperature is fixed to $T=730{\;}^{\circ }$C. The Ga and N fluxes are chosen so that $\phi_{\mathrm{Ga}}/\phi_{N}=0.8$, and the growth rate is ∼0.15 ML/s, i.e., the growth is performed under a slight excess of N.

**Figure 4:**
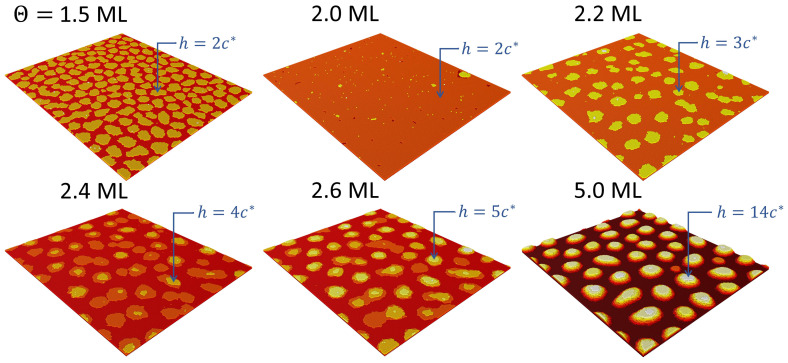
Simulation of the process of growth of 5 ML of GaN on AlN. The various panels show the GaN surface morphology at different instants (corresponding to the specified values of the GaN coverage Θ). The color scale indicating the discrete height is independently chosen in each panel, but the corresponding maximum height is specified in every case.

**Figure 5:**
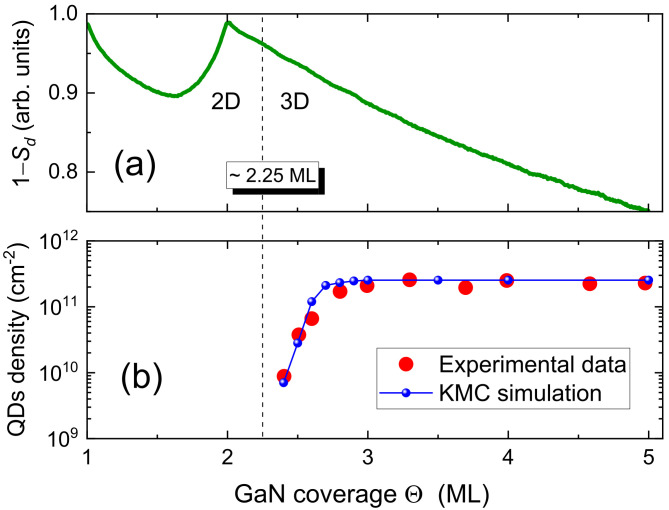
(**a**) Evolution of the step density $1-S_{d}$ as a function of Θ. The vertical line at ≈2.25 ML indicates the critical thickness at which the 2D-3D SK transition occurs. (**b**) QD density as a function of Θ. The results obtained from the KMC simulations are compared with the experimental data of Ref. [21].

Figure 4 shows the evolution of the GaN film morphology as the deposition progresses, from a completely formed first monolayer up to a final GaN coverage of Θ=5 ML. It can be observed that the growth of the second monolayer takes place by the 2D layer-by-layer mode, i.e., by the formation of 1 ML high nuclei that grow progressively into laterally extended 2D islands (platelets) by capturing the adatoms that had been deposited on the surface (see the results in Figure 4 for a coverage of 1.5 ML). These platelets coalesce until the second monolayer is fully completed. However, from that point on the morphology changes drastically: although initially 2D platelets start to grow as before, at a deposition of 2.2–2.4 ML new clusters are formed on top of some of those platelets, and simultaneously, due to the strain effects, the lateral growth of the platelets becomes energetically inconvenient and therefore strongly inhibited before coalescence becomes important. This qualitative change, with the formation of stable 3D islands (2 or more ML high) on top of the flat surface (clearly observed in Figure 4 for a coverage of 2.6 ML), marks the onset of the Stranski–Krastanov transition [21]. In order to quantitatively assess these observations, we show in Figure 5a the evolution of the calculated step density $1-S_{d}$ as a function of Θ. The oscillation between the first and second ML is indicative of the layer-by-layer growth mode. Shortly after the completion of the second ML (identified by the disappearance of the oscillation), the critical thickness is reached at Θ∼2.25 ML, and the growth is changed to the SK mode, with the formation of the first 3D islands (quantum dots), as commented above. The SK transition does not manifest itself very clearly in this representation, although a very small elbow associated to it can be hinted at 2.25–2.4 ML. For further deposition, from 2.2 to 3 ML, the quantity $1-S_{d}$ decreases monotonically, in that part of the process the existing islands continue to grow, while at the same time new 3D islands begin to form. This is best observed in Figure 5b, where we depict the evolution of the QD density with increasing GaN coverage. The simulation results demonstrate that the QD density increases rapidly from the onset of the SK transition until approximately 3.0 ML. From that moment on and up to 5 ML, the number of QDs remains constant, and the growth continues exclusively by increasing the volume of the existing QDs, as can be observed in Figure 4. For the sake of comparison, we also present in Figure 5b the corresponding experimental results of the QD density reported in Ref. [21]. We conclude that the results from the numerical simulations concerning the different stages of the growth process and the overall features of the quantum dot ensemble are generally in good agreement with the growth experiments. There remain, however, some discrepancies that could be addressed in further work. Most noticeably, the experiments demonstrate the appearance, with increasing coverage after density saturation, of a bimodal height distribution, with fixed height islands coexisting with larger islands ever growing in size. Our present model does not reproduce such behavior.

### 3.3. QD Growth as a Function of $\phi_{\mathrm{Ga}}/\phi_{N}$ Ratio

One of the factors easily controllable in GaN/AlN heteroepitaxial growth experiments, and known to influence the appearance of the SK transition and the characteristics of the resulting QDs, is the ratio between the nominal fluxes of Ga and N, $\phi_{\mathrm{Ga}}/\phi_{N}$ [18,19,20]. In this section, we use our simulations to study the morphology of the QD layer as a function of $\phi_{\mathrm{Ga}}/\phi_{N}$. The total amount of GaN deposited is Θ=3 ML and the substrate temperature is fixed to $T=730{\;}^{\circ }$C.

**Figure 6:**
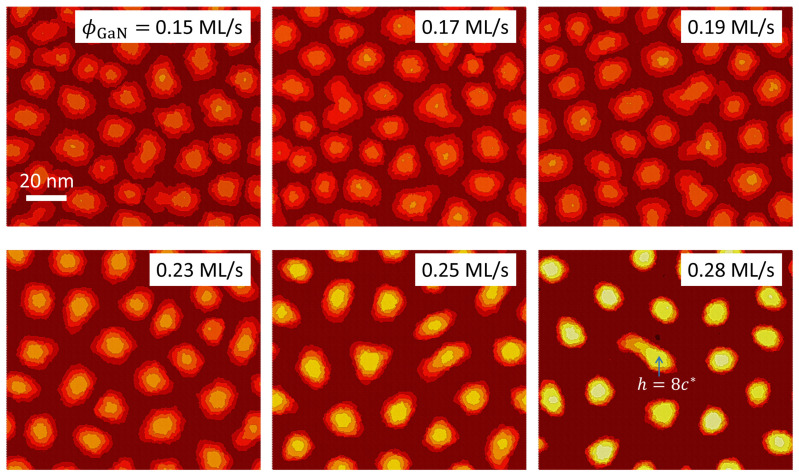
Surface morphology of a GaN epitaxial layer grown on an AlN substrate for simulations with different nominal Ga fluxes $\phi_{\mathrm{Ga}}$ (specified in each panel of the figure). The N flux $\phi_{N}$ has been set at 0.28 ML/s. The color scale indicating the discrete height is the same for all panels. A reference height is provided in the panel for $\phi_{\mathrm{Ga}}=0.28$ ML/s.

Figure 6 shows the film morphologies simulated for values of $\phi_{\mathrm{Ga}}$ between 0.15 and 0.28 ML/s. The N flux $\phi_{N}$ has been set at 0.28 ML/s. As can be observed, for $\phi_{\mathrm{Ga}}$ less than 0.23 ML/s ($\phi_{\mathrm{Ga}}^{\mathrm{eff}}/\phi_{N}\approx 0.88$), the SK transition competes with the kinetically induced formation of extended 2D platelets: in this regime the mobility of the adatoms on the surface, directly dependent on the ratio $\phi_{\mathrm{Ga}}^{\mathrm{eff}}/\phi_{N}$, is not high enough to promote the nucleation of 3D precursor islands well defined and separated from each other. Therefore, the system partially relaxes its elastic energy by means of a rough surface instead of through a genuine 2D-3D transition. This behavior generally agrees with that observed in growth experiments [18,19]. Furthermore, for simulations with increasing $\phi_{\mathrm{Ga}}/\phi_{N}$ beyond $\phi_{\mathrm{Ga}}\geq 0.23$, we observe a significant increase in the average island height, along with a slight decrease in the diameter, together with a progressive size homogenization. The origin of this trend lies in the decrease in both the diffusion barrier $E_{\mathrm{dif}}^{\left(0\right)}$ and the binding energy $E_{b}$ (that can be associated to an increase in the effective mobility of the adatoms) as $\phi_{\mathrm{Ga}}$ increases, so that the diffusion of the GaN adatoms across the surface is predominantly determined by the inhomogeneities in the elastic energy. Therefore, the precursor 2D islands slow down their lateral growth earlier than for growth under lower $\phi_{\mathrm{Ga}}/\phi_{N}$, and the SK transition to 3D growth sets is clear. Our calculations (not shown) also demonstrate that for Ga fluxes beyond 0.23 ML/s, the QD density decays linearly as we get closer to the stoichiometry (which corresponds to $\phi_{\mathrm{Ga}}=\phi_{N}+\delta \phi_{\mathrm{GaN}}∼0.3$ ML/s). Simulations carried out considering a higher N flux of value $\phi_{N}=0.34$ ML/s (not shown here) reveal a tendency of the GaN layer to relax its elastic energy via the formation of irregular islands of small height and large diameter (instead of fully developed 3D dots), with essentially constant density.

### 3.4. QD Growth as a Function of Substrate Temperature

We have observed that variations in the mobility of the adatoms, as a function of $\phi_{\mathrm{Ga}}/\phi_{N}$, can affect the growth (for a fixed temperature). On the other hand, since the surface diffusion is a thermally activated process, significant changes in adatom mobility can in principle be achieved by varying the temperature. It is relevant, therefore, to study in detail the growth of QDs as a function of the substrate temperature. To that end, we have performed simulations at various temperatures *T* between $610{\;}^{\circ }$C and $760{\;}^{\circ }$C. The total amount of GaN deposited is fixed to 3 ML. The fluxes have been set to $\phi_{N}=0.34$ ML/s and $\phi_{\mathrm{Ga}}=0.25$ ML/s, respectively. However, the nominal Ga flux $\phi_{\mathrm{Ga}}$ has been modified when necessary (for $T>730{\;}^{\circ }$C) so as to compensate the increment of desorption with temperature and thus maintain an approximately constant value of the growth rate around ∼0.25 ML/s. This allows one to ensure that the characteristics of the QD formation and growth will depend exclusively on the adatom mobility activated by the temperature.

**Figure 7:**
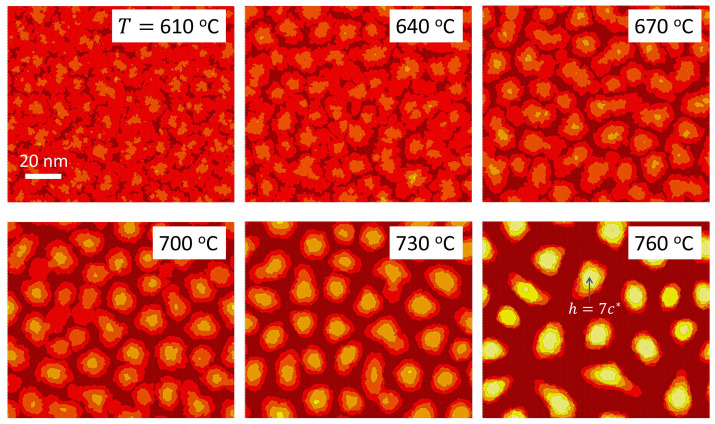
Surface morphology of a GaN epitaxial layer grown on an AlN substrate as obtained by simulations performed at different temperatures (specified in each panel of the figure). The color scale indicating the discrete height is the same for all panels. A reference height is provided in the panel for $T=760{\;}^{\circ }$C.

**Figure 8:**
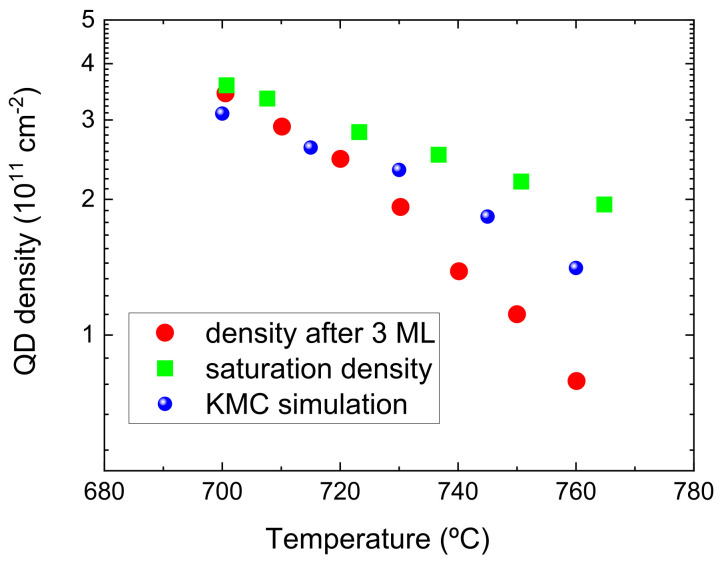
QD density obtained from the KMC simulations as a function of temperature (blue circles). For comparison, we also display two sets of experimental densities (red circles and green squares) measured in Ref. [21] (see text).

Figure 7 shows the different GaN layer morphologies obtained at various temperatures. The first thing to notice is the absence of the SK transition for temperatures below $700{\;}^{\circ }$C, attributed to the insufficient mobility of the adatoms. This finding agrees qualitatively with the experiments of Ref. [21], where it is reported that the formation of QDs is suppressed for temperatures below $680{\;}^{\circ }$C. The discrepancy in the temperature values can be tentatively attributed to differences in other growth conditions between the simulation and experiment, such as the N flux used, which is not specified in the report of the experiments. The simulations also demonstrate that, as we increase the temperature from $700{\;}^{\circ }$C, there is a reduction in the diameter of the QDs. This is accompanied by a clear decrease in the QD density. This trend is best illustrated in Figure 8, which shows the values of the QD density extracted from the simulations, together with experimental results from Ref. [21]. As we have observed above, the island density tends to saturate at sufficiently high GaN coverage. The experiments demonstrate that this saturation density depends on the substrate temperature *T*, but when $T>730{\;}^{\circ }$C saturation occurs for GaN coverages larger than 3.0 ML. Therefore, two different sets of experimental density values are displayed in the figure: one corresponding to a fixed GaN coverage of 3 ML (as in the simulations) and the other corresponding to the saturation density. The simulation results exhibit a decrease in the island density with increasing temperature that lies between the two sets of experimental results. This can be considered a good agreement on account that the growth conditions established in the simulator may not be exactly the same as those used in the experiment, as commented above.

### 3.5. Growth of Stacks of GaN/AlN QDs: Correlation Effects

The previous results have confirmed the utility of the model developed to investigate the self-assembling process of a single layer of GaN QDs under different growth conditions. In this section, we will show simulations of the growth of stacks of GaN/AlN QD layers. For the modeling of the stacks, we proceed as follows: once the simulation of the first GaN layer is finished, it is assumed that it is covered with an AlN layer (spacer) with a chosen thickness *d*. This way, we obtain a new flat AlN surface on which the growth of the next GaN layer can proceed. However, due to the presence of QDs embedded in the AlN substrate, the surface will exhibit a nonuniform strain. This strain, calculated by means of Eshelby’s inclusion theory [50,51], allows one to obtain the local AlN lattice parameters on the free surface, which are used as input parameter in the calculation of the elastic energy during the growth of the next QD layer. The process is repeated up to the completion of the total number of QD layers. When the simulation of the last layer is finished, the system is covered with an AlN capping layer having the same thickness as the spacers. The above procedure allows us to compute the strain energy density $U\left(x\right)$ throughout the entire volume of the heterostructure. This detailed description of the strain, which has not been incorporated before in KMC growth simulations of GaN QD multilayers, is, however, very useful to analyze the elastic interaction between the QDs through the barrier and its dependence on the spacer thickness.

**Figure 9:**
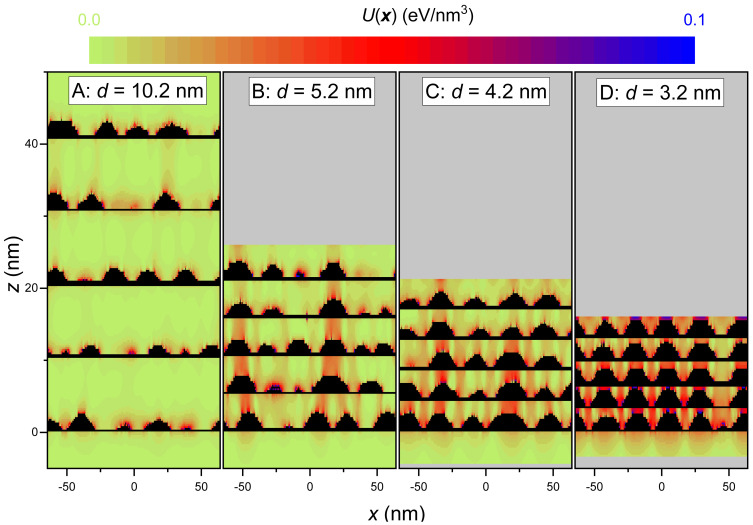
Vertical cross sections through the simulated stacks consisting of five QD layers separated by different spacer thicknesses: d=10.2 nm (stack **A**), d=5.2 nm (**B**), d=4.2 nm (**C**), and d=3.2 nm (**D**). For each stack, the distribution of the elastic energy density U(x) is shown by means of a pixelated color map. The color scale has been truncated at 0.1 eV/nm${}^{3}$, so that most the GaN volume, which is strained beyond that value, appears as black colored.

In the simulations, a total amount of 5 ML is deposited in each GaN layer. The growth conditions were fixed at $T=730{\;}^{\circ }$C and $\phi_{\mathrm{Ga}}=0.25$ ML/s and $\phi_{N}=0.31$ ML/s. Although real stacks can contain tens and even hundreds of layers [23,24,54], in this work, due to computer memory limitations, we consider stacks of five QD layers each. We present simulations of four stacks with different spacer thicknesses, labeled A ($d=41c^{*}=10.2$ nm), B ($d=21c^{*}=5.2$ nm), C ($d=17c^{*}=4.2$ nm) and D ($d=13c^{*}=3.2$ nm). Figure 9 depicts, next to each other, random cross sections of the four stacks. In the stack A, the different layers seem to be totally independent. There is a great dispersion in sizes and a total absence of vertical correlation. As the spacer thickness is decreased, it is already grasped in stack B a certain tendency for the dots in successive layers to grow above each other, but an important dispersion of sizes is still present. However, for stack C (spacing d=4.2 nm) a clear trend for the QDs to nucleate just above the QDs of the preceding layers is detected. This behavior is more pronounced for the stack D (spacing d=3.2 nm) where the spatial correlation in the vertical direction and the effect of homogenization of sizes and density is evident. This behavior should be even more pronounced with further increasing the number of layers, as is observed in real stacks [23]. The figure also displays color maps of the elastic energy density for the whole of the structure. It is apparent that the accumulation of (tensile) strain energy in the regions of the AlN spacer above the buried dots can act as a driving factor for the aligned growth of the QDs in the following layers.

## 4. Discussion

We have developed a (3D) lattice kinetic Monte Carlo model for the simulation of the heteroepitaxial growth of GaN/AlN systems. The species simulated are effective GaN adatoms. The model is standard in that it includes the relevant processes of deposition, diffusion and desorption, and implements the BKL algorithm. In addition, it incorporates several special characteristics that we highlight in the following. On account of the usual wurtzite crystalline structure of the III-N semiconductors, the simulation domain is built on an hexagonal lattice. The influence of the elastic strain inherent to lattice-mismatched growth is incorporated through an inhomogeneous correction $E_{\mathrm{str}}\left(I\right)$ to the diffusion and desorption energy barriers. This quantity defined at the lattice sites is related to the strain energy density at the surface of the growing GaN film $U(x_{⊥})$. The latter quantity is in turn calculated within linear elastic continuum theory by an efficient procedure purposely devised and based on the combined use of the Eshelby inclusion concept and the Green’s function method. This procedure accounts for the elastic anisotropy of the system and allows to dynamically account for the deformation at the free surface of the irregular profile of the growing film. The above strain energy considerations supersede previous works on similar topics, which make drastic simplifications in various respects [8,9,55]. On the other hand, our goal to study the self-organization effect imposes the need to perform simulations over a large area domain on long time scales. In order to improve the computational speed so as to fulfill the above requirements, we have introduced various modifications to the standard BKL algorithm. The most important one has been to allow the isolated adatoms to perform longer discrete jumps than the laterally bound adatoms (multiscale kinetic Monte Carlo algorithm). The direction of these jumps has been selected according to the strain landscape in which they take place. With these improvements, efficient realistic simulations on surfaces with side lengths close to one hundred nanometers, over time intervals of tens of seconds (multiplied by up to 5 in the case of stacks) are possible. We note that our model assumes a flat substrate surface. In practice, most often the substrates present some degree of deviation from flatness. For example, in the experiments of Ref. [21] to which we compare our simulations, the AlN surface is characterized by about 30 nm wide terraces and spiral hillocks. This structure however does not seem to affect the nucleation of the dots which are distributed rather homogeneously. In some other works, vicinal substrates with an intentional miscut are used, resulting in a step organization, made of regularly ordered terraces, at the AlN surface [56]. When the terrace width is larger than the diffusion length of the adatoms, their kinetics is essentially equal to that in a flat surface. This leads to a homogeneous nucleation, with dots covering the entire surface. On the contrary, when the diffusion length is comparable to the width of the terraces, the GaN nucleation at steps is energetically favorable, leading to a clear alignment of the dots along the AlN steps. The modeling of this specific behavior is interesting and could motivate future extensions of our KMC model.

Let us now comment on what we consider the main limitations of the described KMC model. In the first place, we note that it is not strictly atomistic since the simulated units are effective GaN adatoms. This means that the parameters are also effective and, in principle, their values must be obtained by trial and error. However, in our case we have developed a systematic strategy to ascertain the optimal values adapted to our purposes. Initially, simulations of homoepitaxial GaN PA-MBE growth have been quantitatively compared with corresponding available experiments. This comparison has allowed one to estimate the intrinsic parameters governing the growth dynamics on the GaN surface in the N-rich regime, and its dependence on the nominal fluxes. Afterwards, the strain-related parameters, notably the parameter *k* commented below, have been fixed to achieve agreement with some key experimental results, such as the SK critical thickness, and the correlation spacing in QD stacks. The other noteworthy point of the model is the introduction of the free parameter *k* in Equation (5). As mentioned above, in the case of isolated adatoms we have replaced the short jumps with longer jumps, which allow us to considerably speed up the simulations. However, in doing this we are eliminating the cumulative effect of the elastic inhomogeneities after many short jumps. To compensate for it, we have added a bias in the direction of the long jumps. This bias is proportional to the difference between the elastic energies of the initial and arrival sites and to the number of lattice sites between them. The free parameter *k* does not alter the surface distribution of $E_{\mathrm{str}}\left(x_{⊥}\right)$ calculated by the elastic continuum theory but just magnifies the amplitude of the strain-induced modulation between the initial and final sites to compensate for the loss of the cumulative effect of many short random jumps. A similar parameter has been introduced in the KMC simulations of Ref. [9]. It is remarkable that a single parameter *k* is sufficient for obtaining a reasonably good correspondence with the experimental data, as commented below.

The capabilities of the developed KMC model have been demonstrated by comparing its simulations to the comprehensive growth experiments of Ref. [21]. In particular, the influence of macroscopic growth parameters such as the surface coverage, the deposition fluxes and the substrate temperature has been investigated. We summarize here the main findings obtained in the simulations that are in qualitative (and even quantitative in many instances) agreement with the experiments: (i) The KMC simulations demonstrate clearly that the GaN growth passes four stages: (1) initially, the growth is layer by layer; (2) subsequently, 2D precursor islands form, which (3) later transform into genuine 3D islands through the SK transition; (4) during further GaN growth, the 3D islands increase their size, whereas their density remains constant. (ii) Within the model the adatom mobility, which is coded through the diffusion rate, depends directly on the temperature and indirectly on the impinging material fluxes. The simulations have demonstrated that these parameters (and therefore the adatom mobility) strongly influence the SK transition. This is in agreement with the experiments where it is observed that the surface morphology of GaN layers grown by PA-MBE depends crucially on the Ga/N flux ratio and the substrate temperature. Finally, we have presented simulations of the growth of stacked GaN QD layers separated by AlN spacers, fully taking into account the elastic strain field in the multilayer system. The study as a function of the spacer layer thickness reveals the important role of the in-plane lattice parameter modulation in each spacer layer surface (induced by the buried QDs) in directing the vertical alignment of the dots, together with their size and density homogenization. Again, the simulations yield results in good qualitative agreement with experimental data concerning the observed vertical correlations [23]. Still, the model fails to reproduce some features clearly observed in the experiments. The most notable is the bimodal distribution of sizes observed in these systems under certain growth conditions [21]. On the other side, although the simulations results for the QD height and diameter are compatible with those reported in the experiments, they do not clearly reproduce the typical hexagonal base pyramid shapes found in real systems of self-assembled GaN QDs [17]. This is probably due to the lack of an explicit atomistic description of the GaN wurtzite structure.

In summary, the KMC model developed here for the simulation of strained heteroepitaxial growth can be considered as an excellent compromise between continuum models [57] and fully atomistic simulations [58,59]. We note that, even though the model has been tailored to the study of PA-MBE growth of the GaN/AlN system under N-rich conditions, the general set up can be modified or extended to have wider applicability. In this respect, it could be easily adapted, e.g., to deal with the growth of GaN quantum dots on non-polar surfaces [25].

## Figures and Tables

**Figure A1:**
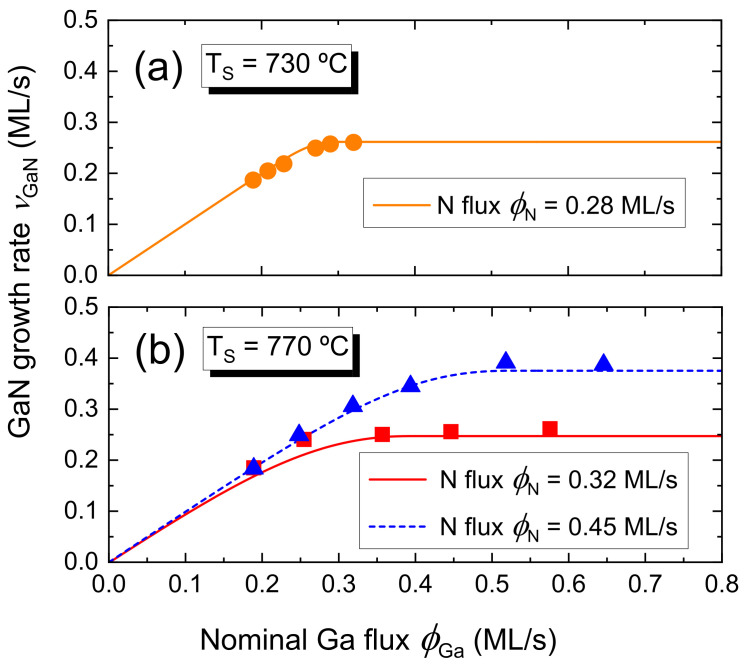
Growth rate of GaN as a function of the impinging Ga flux, for two substrate temperatures and two impinging N fluxes. The dots of (**a**,**b**) are experimental data taken from Refs. [20,53], respectively. The lines represent fits calculated within the framework of the model described in the text.

## Data Availability

Not applicable.

## Abbreviations

The following abbreviations are used in this manuscript:

KMC	Kinetic Monte Carlo
SK	Stranski–Krastanov
QD	Quantum dot
PA-MBE	Plasma-assisted molecular beam epitaxy
ML	Monolayer
BKL	Bortz–Kalos–Lebowitz

## Appendix A. Model for the Effective Flux $\phi_{\mathrm{Ga}}^{\mathrm{eff}}$

We propose a simple model for the effective flux $\phi_{\mathrm{Ga}}^{\mathrm{eff}}$ that incorporates the influence of the thermal decomposition of GaN. If we would only take into account the Ga evaporation, the net Ga flux should be less than the nominal one leaving the Knudsen cell. This effect can be represented by a sticking factor α (α∈[0,1]), which is the fraction of Ga atoms impinging on the surface that will not evaporate and remain on the surface.

However, within the growth temperature range relevant here, the decomposition of GaN previously formed on the surface represent an extra source of Ga that must be incorporated into the global calculation of the effective flux. The rate of GaN decomposition is the thermal decomposition rate of GaN in the vacuum, $\delta \phi_{\mathrm{GaN}}$, which can be described as an Arrhenius type function:

(A1) $$\delta \phi_{\mathrm{GaN}}=\nu_{\mathrm{GaN}}e^{-\frac{E_{\mathrm{GaN}}}{k_{B}T}}\;,$$

whose parameters, $\nu_{\mathrm{GaN}}=6.3\times 10^{13}\;s^{-1}$ and $E_{\mathrm{GaN}}=3.1\;\mathrm{eV}$, are taken from Ref. [53].

In our model, we start from the above observations and define the growth rate of GaN as:

(A2) $$\nu_{\mathrm{GaN}}=\alpha \phi_{\mathrm{Ga}}-\delta \phi_{\mathrm{GaN}}+\alpha \delta \phi_{\mathrm{GaN}}\;.$$

The term $\delta \phi_{\mathrm{GaN}}$, given by (A1), represents the correction to the growth rate due to the decomposition of GaN, whereas $\alpha \phi_{\mathrm{Ga}}$ is the net flux of deposited Ga that remains on the surface without evaporating, and $\alpha \delta \phi_{\mathrm{GaN}}$ is the extra Ga flux resulting from the GaN decomposition which, given the N-rich environment, will remain on the surface. The effective Ga flux on the surface is therefore:

(A3) $$\phi_{\mathrm{Ga}}^{\mathrm{eff}}=\alpha \left(\phi_{\mathrm{Ga}}+\delta \phi_{\mathrm{GaN}}\right)\;.$$

The coefficient α itself depends in general on the nominal fluxes $\phi_{\mathrm{Ga}}$ and $\phi_{N}$. We assume this dependence to follow a distribution type function with the form:

(A4) $$\alpha \left(\phi_{\mathrm{Ga}},\phi_{N}\right)=A+\frac{1-A}{1+e^{B\left(\phi_{\mathrm{Ga}}-\phi_{N}-\delta \phi_{\mathrm{GaN}}\right)}}\;.$$

In order to determine the parameters *A* and *B*, we focus on the stoichiometry condition at the surface which, for a given nominal N flux $\phi_{N}$, is reached by increasing the nominal Ga flux up to a value $\phi_{\mathrm{Ga}}^{\left(s\right)}$ equal to $\phi_{N}$ plus the correction corresponding to the Ga desorption rate that, in these conditions, coincides with the GaN decomposition rate:

(A5) $$\phi_{\mathrm{Ga}}^{\left(s\right)}=\phi_{N}+\delta \phi_{\mathrm{GaN}}\;.$$

At the stoichiometry situation, the growth rate reaches the value:

(A6) $$\nu_{\mathrm{GaN}}^{\left(s\right)}=\phi_{N}-\delta \phi_{\mathrm{GaN}}\;.$$

Substituting (A6) and (A5) into (A2), we obtain an expression for the coefficient α in stoichiometry conditions:

(A7) $$\alpha^{\left(s\right)}=\frac{\phi_{N}}{\phi_{N}+2\delta \phi_{\mathrm{GaN}}}\;.$$

Now, by requiring $\alpha \left(\phi_{\mathrm{Ga}}^{\left(s\right)},\phi_{N}\right)=\alpha^{\left(s\right)}$, we determine *A*:

(A8) $$A\left(\phi_{N}\right)=\frac{\phi_{N}-2\delta \phi_{\mathrm{GaN}}}{\phi_{N}+2\delta \phi_{\mathrm{GaN}}}\;.$$

Then, by imposing the condition that the growth rate is maximum in stoichiometry conditions, i.e., ${\left(\frac{\partial \nu_{\mathrm{GaN}}}{\partial \phi_{\mathrm{Ga}}}\right)}_{\phi_{\mathrm{Ga}}^{\left(s\right)}}=0$, the parameter *B* is found to be:

(A9) $$B\left(\phi_{N}\right)=\frac{\phi_{N}}{\delta \phi_{\mathrm{GaN}}\left(\phi_{N}+2\delta \phi_{\mathrm{GaN}}\right)}\;.$$

Finally, with the coefficient α determined by the above procedure, the effective Ga flux $\phi_{\mathrm{Ga}}^{\mathrm{eff}}$ can be obtained by means of (A3) for any growth condition within the N-rich regime. In order to test the validity of our model, we have compared in Figure A1 our results for $\nu_{\mathrm{GaN}}$ as a function of $\phi_{\mathrm{Ga}}$ with the growth rates measured experimentally in Refs. [20,53]. An excellent agreement with the experiments is found even at temperatures as high as $770{\;}^{\circ }$C. This shows that, at least up to this temperature, the desorption of N can be considered negligible.
